# The SAIL databank: linking multiple health and social care datasets

**DOI:** 10.1186/1472-6947-9-3

**Published:** 2009-01-16

**Authors:** Ronan A Lyons, Kerina H Jones, Gareth John, Caroline J Brooks, Jean-Philippe Verplancke, David V Ford, Ginevra Brown, Ken Leake

**Affiliations:** 1 Health Information Research Unit (HIRU), Centre for Health Information Research & Evaluation (CHIRAL), School of Medicine, Swansea University, Swansea, Wales, UK; 2 Health Solutions Wales (HSW), Brunel House, Cardiff, Wales, UK

## Abstract

**Background:**

Vast amounts of data are collected about patients and service users in the course of health and social care service delivery. Electronic data systems for patient records have the potential to revolutionise service delivery and research. But in order to achieve this, it is essential that the ability to link the data at the individual record level be retained whilst adhering to the principles of information governance. The SAIL (Secure Anonymised Information Linkage) databank has been established using disparate datasets, and over 500 million records from multiple health and social care service providers have been loaded to date, with further growth in progress.

**Methods:**

Having established the infrastructure of the databank, the aim of this work was to develop and implement an accurate matching process to enable the assignment of a unique Anonymous Linking Field (ALF) to person-based records to make the databank ready for record-linkage research studies. An SQL-based matching algorithm (MACRAL, Matching Algorithm for Consistent Results in Anonymised Linkage) was developed for this purpose. Firstly the suitability of using a valid NHS number as the basis of a unique identifier was assessed using MACRAL. Secondly, MACRAL was applied in turn to match primary care, secondary care and social services datasets to the NHS Administrative Register (NHSAR), to assess the efficacy of this process, and the optimum matching technique.

**Results:**

The validation of using the NHS number yielded specificity values > 99.8% and sensitivity values > 94.6% using probabilistic record linkage (PRL) at the 50% threshold, and error rates were < 0.2%. A range of techniques for matching datasets to the NHSAR were applied and the optimum technique resulted in sensitivity values of: 99.9% for a GP dataset from primary care, 99.3% for a PEDW dataset from secondary care and 95.2% for the PARIS database from social care.

**Conclusion:**

With the infrastructure that has been put in place, the reliable matching process that has been developed enables an ALF to be consistently allocated to records in the databank. The SAIL databank represents a research-ready platform for record-linkage studies.

## Background

Recent years have seen a huge growth in the development of electronic systems to capture individual records in the course of health and social care service delivery [1]. These routinely-collected data have enormous potential in health-related research, quality improvement, service planning and enhanced clinical decision-making [2], and such information could revolutionise health research if longitudinal individual health records can be developed from existing systems or through new developments [3].

The Health Information Research Unit (HIRU) is an initiative developed by the School of Medicine at Swansea University. It is core-funded through the Wales Office of Research & Development as part the Welsh Assembly Government's commitment to the UK Clinical Research Collaboration (UKCRC) [4]. The main aim of HIRU is to realise the potential of electronically-held, person-based, routinely-collected information for the purpose of conducting and supporting health-related research. HIRU has set up the SAIL (Secure Anonymised Information Linkage) databank to bring together and link the widest possible range of anonymised person-based data, and has done this using a split-file approach to anonymisation to overcome the confidentiality and disclosure issues in health-related data warehousing. Through this method, datasets being provided to the SAIL databank are split at the source organisation into demographic data and clinical data. A system linking field is used to ensure the data can be re-joined later. The demographic data comprises the commonly-recognised person-based variables of first name, surname, gender, date of birth and postcode. The clinical data covers data such as diagnostic tests, therapeutic procedures and interventions, and these data are transferred directly to HIRU. The demographic data are transferred to Health Solutions Wales (HSW) [5] for pseudonymisation and the allocation of an Anonymous Linking Field (ALF) to each record in place of the demographic data. An ALF takes the form of a unique 10-digit number assigned to each individual in a dataset. This product is transferred to HIRU where it is joined to the clinical data via the system linking field [6].

Although the SAIL data are anonymised and encrypted, it is essential that the capability to link the data at the individual record level be retained if they are to be useful in health research. Linkage is necessary for a variety of reasons, including: to allow links within and between databases from different sources; to ensure comparisons are meaningful; to assess the completeness of recruitment to research studies; to allow inequalities in health and wider factors (such as social issues) to be investigated; to validate research findings; and to enhance patient follow-up and adverse event reporting in clinical trials [7-9]. Successful record linkage is dependent on the presence of specific variables in the dataset that can reliably be used in the matching process to assign a consistent identifier for each individual. In some cases an exact match can be created, providing deterministic record linkage (DRL). However, it is more usual in complex datasets that some values are missing, and that unique identifiers are not present for all, if any, records. In these cases probabilistic record linkage (PRL) methods are used, taking account of the probabilities of agreement and disagreement between a range of matching variables [10,11]. Because of this, PRL tends to have a higher sensitivity, but a lower specificity than DRL [12].

In the UK, health and social care are provided by multiple agencies using disparate database systems. There is no system of unique national identity number, but all persons registered with the National Health Service (NHS) in England and Wales are assigned a unique 10-digit NHS number, and this is used as the personal identifier for patients across different NHS organisations [13]. As well as this, the regularly maintained NHS Administrative Register (NHSAR) which comprises details of everyone who has registered or accessed health services in Wales, can be used as a proxy for a Welsh-population database. It contains identifying information such as name, address (and historical addresses), postcode, gender, date of birth, general practice of registration and the NHS number.

Having established the infrastructure of the SAIL databank [6], the aim of the study described here was to implement an accurate matching process to enable the assignment of an ALF to person-based records so that the databank is ready for record-linkage research studies.

## Methods

### Questions to be addressed

Methods were devised to address two questions. The first question assessed the accuracy of accepting the NHS number supplied in routine NHS data as the basis of a unique identifier. The second question assessed the effect on numbers of records matched of varying the techniques applied in matching each of three different datasets to the NHSAR. As this study involved work with potentially person-identifiable variables it was conducted in Health Solutions Wales (HSW) who act as the Trusted Third Party (TTP) in providing HIRU with a data pseudonymisation service [6].

### Datasets

Three test datasets of person-based records from the health economy of Swansea were used in this study. These were: a primary care dataset from across the general practices (GP) in the area; a secondary care dataset of hospital in-patient data from the Patient Episode Database for Wales (PEDW); and a local authority social services dataset called the PARIS system. The PARIS system is an electronic record of individuals receiving various social services including, mental health, learning disabilities and elderly care under the auspices of the local authority. These will be referred to as the GP dataset, the PEDW dataset and the PARIS dataset, respectively. As part of NHS primary and secondary care services, the GP and PEDW datasets are structured to include an NHS number. The PARIS database, as part of social services, does not contain NHS numbers. The criteria used to assess matching efficacy were: forename, surname, gender, postcode of residence and date of birth. These will be referred to as the set of matching variables. The NHSAR was used as the reference dataset and records in the test datasets would be expected to have a match on the NHSAR.

### Matching algorithm

The MACRAL (Matching Algorithm for Consistent Results in Anonymised Linkage) algorithm was developed for the work of HIRU. MACRAL is an SQL-based algorithm that is used to apply DRL and PRL methods to the set of matching variables. DRL looks for an exact match on all five variables. The probability-based linkages make use of a variety of techniques, including some which allow similar but not identical query strings to be accepted as possible matches [14]. These include Lexicon matching and Soundex matching. The Lexicon used in this study is a Welsh-specific list of alternative forenames, based on variants in the registered name given by persons listed on the NHSAR, such as Betty, Elsie, Liz, etc. for Elizabeth. Soundex matching is a standard technique that uses codes for variant phonetic spellings of the forename or surname. Probabilities are assigned to the match success, and these are based on likelihood ratios calculated using a Bayesian approach of prior and posterior odds, by taking into account the distributions of the set of variables on the NHSAR for the Welsh population. For example, it takes into account the occurrence of common surnames, such as Jones, in deriving the likelihood ratio to create the weighting assigned to the match. It also recognises the non-independence of certain pieces of information, such as the male gender and recognised male first names, in generating the likelihood ratio.

The posterior odds are calculated as:

Posterior odds = prior odds * likelihood ratio

The likelihood ratios are calculated as follows:

Firstly, where the demographic variables match (e.g. on surname) -

$$\text{Likelihood ratio}=\frac{p(\text{match|records relate to the same person})}{p(\text{match|records relate to a different person})}$$

And where the demographic variables do not match -

$$\text{Likelihood ratio}=\frac{p(\text{non-match|records relate to the same person})}{p(\text{non-match|records relate to a different person})}$$

In this way, pairs of variables found to match increase the odds of a match and pairs of variables that don't match decrease the odds. This is applied to each of the set of five variables for each record to produce the final cumulative probability of a match. Acceptable matching thresholds for a given dataset can be specified as required. A range of matching probabilities with cut off points of 99%, 95%, 90% and 50% were assessed for each of the three datasets in this study.

### Assessing the accuracy of NHS numbers in routine data

This was addressed by matching each of two NHS datasets against the NHSAR on the set of matching variables, and using the results to allocate an NHS number to the records in those datasets. These were a GP dataset and a PEDW dataset. A GP dataset of registered patients (n = 229,127) was extracted for this study, and of this, the sub-set of 229,117 records with a valid NHS number was used. A sub-set of the PEDW data was used to ensure manageable computations, and it was arbitrarily set as records with admission dates on the 15th of every month from 1998–2007 (n = 290,650). Of this sub-set, records with a valid NHS number were used to develop a test dataset (n = 264,868). The resulting GP and PEDW datasets included the set of matching variables. Supplied NHS numbers were validated by using the NHS check digit algorithm [15]. DRL and PRL methods were applied to the set of variables to allocate an NHS number to the records in the GP and PEDW datasets. The degree of agreement between the allocated NHS number and the NHS number supplied in the dataset was checked and used to calculate specificity and sensitivity values. These are defined respectively in this context as: number of matches found to correspond to the same NHS number in the GP or PEDW dataset, and total number of matches made. Where this process resulted in disagreement this was taken as an error in the matching process or in the GP or PEDW dataset, as this work uses the assumption that the reference dataset (the NHSAR) is 100% accurate. There are four possible outcomes in the record matching process: true positive (correct match), false positive (mis-match), true negative (no link present) and false negative (link missed) [16]. However, as all analyses were conducted on anonymised data it was not possible for us to check the actual source of any error, which could be done by reviewing individual clinical notes. This is a limitation of the study and we aim to address this issue in the future so that we can differentiate between the sources of error.

### Varying the probability threshold and optimising the matching technique

The second question measured the impact, on the numbers of records that could be matched, of adopting different probability thresholds and techniques. Cut-off points of 99%, 95%, 90% and 50% were used for each of the three datasets. The GP dataset of registered patients (n = 229,127) extracted for this study was used in full (i.e. including the records without a valid NHS number). Registered patients were chosen to ensure that they were resident in the area and therefore expected to be included on the NHSAR. The PEDW dataset described earlier, including the records without a valid NHS number (n = 290,650), was used. Finally, the assessment was conducted on the PARIS dataset. The numbers on this system are much smaller than on many NHS systems and the entire database (n = 18,540) was anonymised and matched with the NHSAR to assess what proportion of records could be linked to a unique individual within the NHSAR. In each case, the numbers of records in agreement with the NHSAR were taken as successful matches and those resulting in disagreement as error (as previously). However, error rates are not quoted in this case, as the datasets included records without a valid NHS number, and the NHS number is used as the cross-check to calculate the error rate.

## Results

### Assessing the accuracy of NHS numbers in routine data

The initial question assessed the level of accuracy that could be obtained by using a valid NHS number as the basis of an anonymous identifier in routine data. Table 1 shows the results of comparing the NHS number supplied in the GP and PEDW datasets with the NHS number allocated via PRL & DRL methods. The level of agreement between supplied and allocated NHS number was high with disagreement (error) levels of < 0.2%. DRL produced the lower disagreement level, as would be expected with higher specificity, but PRL enabled the greater proportion of records to be linked.

**Table 1:** Assessing the accuracy of NHS numbers in routine data.

Data Source	Type of Record Linkage	Result of comparing the NHS number allocated by the record linkage process with the original submitted NHS number					
		
		Same	Different	Not found	% Agreement	% Disagreement	% Linked
		
		Allocated NHS number **equals **the submitted NHS number	Allocated NHS number **differs **to the submitted NHS number	An NHS number is **not found **by the record linkage process	Of the records that were allocated an NHS number, the percentage that were allocated an NHS number equal to the NHS number submitted	Of the records that were allocated an NHS number, the percentage that were allocated an NHS number different to the NHS number submitted	Of the records that were processed, the percentage that were allocated an NHS Number
		
		a	b	c	= a/(a+b)	= b/(a+b)	= (a+b)/(a+b+c)
Primary Care Practice Clinical Systems (GP) (n = 229,117)	DRL	223,344	40	5,733	99.982%	0.018%	97.498%
	
	PRL – 99% cut off	227,778	51	1,288	99.978%	0.022%	99.438%
	
	PRL – 95% cut off	228,288	55	774	99.976%	0.024%	99.662%
	
	PRL – 90% cut off	228,479	56	582	99.976%	0.025%	99.746%
	
	PRL – 50% cut off	228,699	61	357	99.973%	0.027%	99.844%

Secondary Care Hospital Admissions (PEDW) (n = 264,868)	DRL	216,062	323	48,483	99.851%	0.149%	81.695%
	
	PRL – 99% cut off	244,692	410	19,766	99.833%	0.167%	92.537%
	
	PRL – 95% cut off	247,865	439	16,564	99.823%	0.177%	93.746%
	
	PRL – 90% cut off	249,024	453	15,391	99.818%	0.182%	94.189%
	
	PRL – 50% cut off	250,155	465	14,248	99.815%	0.186%	94.621%

This shows the level of agreement between NHS numbers supplied in the General Practice (GP) dataset (n = 229,117) and the Patient Episode Database for Wales (PEDW) dataset (n = 264,868) with those allocated by the matching process using by DRL and PRL. The NHS Administrative Register (NHSAR) was used as the reference.

### Varying the probability threshold and optimising the matching technique

The effect of varying the matching probability threshold and technique on the numbers of records that could be matched was assessed for each of the three test datasets and the results of these analyses are summarised in Table 2. The percentage of records in the GP dataset that could be matched to the NHSAR was > 99.99%. Varying the acceptable PRL threshold for record matching had negligible effect on the high proportions matched. For the PEDW data, 91.1% of the sample records contained a valid NHS number and by combining these with DRL, the matching rate was increased to 96.6%. The highest match rate was achieved using the combination of valid NHS numbers, DRL and PRL at the 50% threshold.

**Table 2:** Levels of matched records using a variety of techniques.

	Levels of matched records					
	Primary Care General Practice (GP dataset)		Secondary Care Hospital Admissions (PEDW dataset)		Social Services (PARIS database)	

	Number	%	Number	%	Number	%

Sample size	229,127		290,650		18,540	

Valid NHS Number	229,117	99.996%	264,868	91.13%	-	0.00%

Valid NHS Number plus DRL:	229,123	99.998%	280,729	96.59%	14,158	76.36%

Valid NHS Number plus PRL (99% cut off):	229,125	99.999%	287,572	98.94%	17,095	92.21%

Valid NHS Number plus PRL (95% cut off):	229,125	99.999%	288,186	99.15%	17,431	94.02%

Valid NHS Number plus PRL (90% cut off):	229,125	99.999%	288,424	99.23%	17,553	94.68%

Valid NHS Number plus PRL (50% cut off):	229,125	99.999%	288,670	99.32%	17,639	95.14%

**Overall combining Valid NHS, DRL & PRL (50%):**	**229,125**	**99.999%**	**288,683**	**99.32%**	**17,642**	**95.16%**

**Figure 1:**
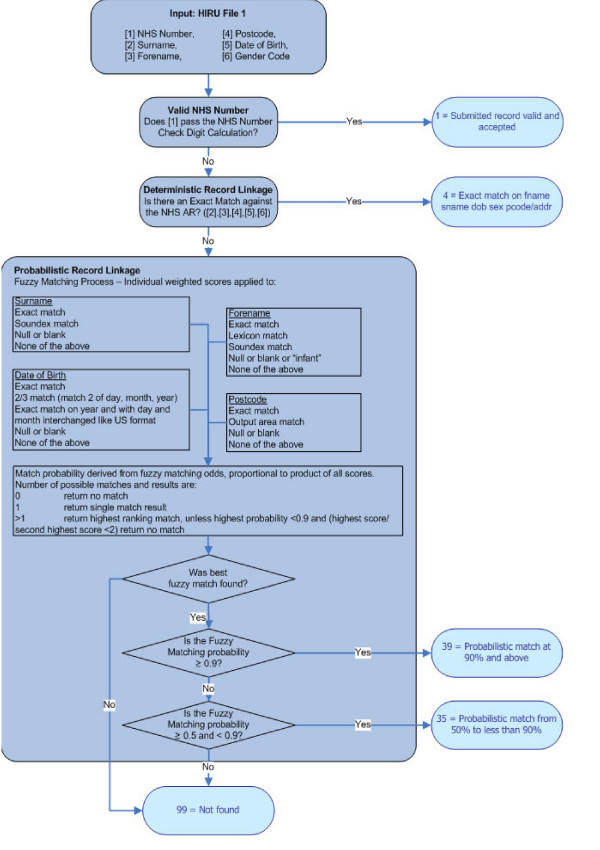
**The matching process conducted via the MACRAL algorithm**. Firstly, records found to have a valid NHS number are accepted. The Matching Algorithm for Consistent Results in Anonymised Linkage (MACRAL) begins with DRL for exact matching on the set of five variables. Following from this, the remaining unmatched records are subjected to PRL methods down to the 50% threshold. Datasets from non-NHS organisations enter the process at DRL.

The numbers (and percentages) of records that could be matched using deterministic record linkage (DRL) and a various thresholds of probabilistic record linkage (PRL) were assessed for each of three test datasets: the GP dataset, the PEDW dataset and the PARIS database. Records with a valid NHS number were accepted. The matching rate achieved by applying DRL followed by PRL (to the 50% threshold) was also assessed, and the final row shows this result of operating the MACRAL algorithm as illustrated in Figure 1.

Of the 18,540 records in the PARIS database, 14,158 (76.4%) were successfully matched to the NHSAR with DRL, with further records being matched using various PRL thresholds. Again the combination of DRL and PRL (50%) yielded the greatest value with 95.2% records matched, leaving a remainder of only 4.8% that could not be matched.

The results obtained from these analyses informed the decision to operate the algorithm in the sequence shown in Figure 1. Firstly, having assessed the accuracy of NHS numbers in routine data and achieving a high degree of agreement with the NHSAR, records with valid NHS numbers are accepted. Next, DRL is carried out on the set of matching variables. Following from this, the remaining unmatched records are subjected to PRL methods down to the 50% threshold. Datasets from non-NHS organisations enter the process at DRL. As a result, an ALF can be allocated to the matched records and this is used as the linking field for each individual in the dataset.

## Discussion

### Assessing the accuracy of NHS numbers in routine data

This assessment confirmed the suitability of accepting a valid NHS number as the basis of allocating the unique identifier: the ALF. The error levels were extremely low, and as would be expected from its greater specificity, were lower for DRL than for PRL. However, PRL with its higher sensitivity resulted in a greater proportion of records being linked than DRL with only slightly higher error rates.

### Varying the probability thresholds and optimising the matching technique

A comparison of probability thresholds and techniques using the GP data resulted in consistently high levels of records matched. As the NHSAR is essentially a list of all patients registered with general practices, whilst some anomalies may occur due to delays in registering new patients, very high levels of NHS number completeness and agreement with the NHSAR were to be expected. This was found to be true for DRL and any variant of PRL with negligible effects on the high proportions matched or on error levels.

High rates of matching were also achieved with the PEDW data demonstrating the notable efficacy of the methods. Although the PARIS database of social services data does not contain NHS numbers, it does contain names, genders, postcodes of residence and dates of birth. It would be expected, therefore, that the matching rates would be considerably lower than were obtained for the NHS datasets. However, using a combination of methods, over 95% of the records were matched. The success of matching on these criteria is a particularly significant result. It means that for datasets, such as these, that originate outside the healthcare sector, the ALF derived from the individual's NHS number (recorded on the NHSAR) can still be consistently applied to their anonymised records. This enables a broad scope for record-linkage studies.

In table 2, the slightly greater numbers of records matched by the sequential process compared to PRL at the 50% threshold are most likely due to rare occurrences of duplicate records on the NHSAR. In those cases, records with an exact match on all five variables would be matched by DRL, but would not be matched by PRL as the highest score/second highest score would be < 2 (as set out in Figure 1).

It is recognised that the increase in sensitivity of lower threshold PRL is accompanied by a decrease in specificity. This increases the risk of acceptance of false positive matches which could have important implications for the analysis of health-related data, particularly if it is to be used to inform clinical practice. As we were unable to distinguish between types of error, we cannot quantify our false positive and false negative rates at this stage. Because of this, the record matching rate is taken into account when extracting data for analysis. The analysis can be repeated including and excluding the records matched at lower PRL thresholds to check for consistency in the results, and to inform the sample that should, therefore, be used for each particular application of the data.

### Comparison with published literature

Record linkage is widely recognised as having far-reaching consequences for the development of innovative approaches to research [3,8]. This study has demonstrated high levels of matching efficacy across three disparate datasets in health and social care that compare favourably with the published literature. For example, specificity values of 100% [17], 99.4% [18], 98% [19] and 89.7% [20], with corresponding sensitivity rates of 92% [17], 99.2% [18], 94% [19] and 99.9% [20] have been reported across various types of record linkage study. The results also compare well with record linkage software packages, such as Link Plus and The Link King [21]. It is worth noting that variations obtained in matching efficacies may be due to the quality and levels of completeness of the datasets as well as to the technical aspects of the linkage systems.

## Conclusion

The matching technique described here has been shown to be a reliable tool to facilitate the allocation of a consistently applied ALF so that record linkage research studies can be conducted on disparate datasets across sectoral boundaries. It should be noted that the development of the MACRAL algorithm was pragmatic, and it has not been compared formally with commercially or publicly available algorithms. However, a comparison with numerous published accuracy and error rates showed similar or slightly better results.

The SAIL databank already holds over 500 million linked-anonymised records. Work is underway to expand the databank in terms of types of dataset, range of data-providing organisations and in geographical coverage. This will encompass broader data than health and social care so that the wider determinants of health can be taken into account. As a result of the infrastructure that has been established and the matching process that has been developed, the SAIL databank represents a research-ready platform for record-linkage studies and a valuable resource for health-related research and service development. Future work will be to carry out an empirical assessment of MACRAL to determine the actual sources of error and to further improve upon the efficacy of the algorithm.

## Abbreviations

ALF: Anonymous Linking Field; MACRAL: Matching Algorithm for Consistent Results in Anonymised Linkage.

## Competing interests

The authors declare that they have no competing interests.

## Authors' contributions

RAL and DVF conceived and designed the study. GJ designed the matching algorithm. J-PV, GB, KL, GJ and CJB carried out the technical work and the analyses. KJ drafted the manuscript. All authors contributed to the manuscript and to the interpretation of the findings, and approved the final manuscript.

## Pre-publication history

The pre-publication history for this paper can be accessed here:

http://www.biomedcentral.com/1472-6947/9/3/prepub
